# Geometry of Quantum Computation with Qutrits

**DOI:** 10.1038/srep02594

**Published:** 2013-09-05

**Authors:** Bin Li, Zu-Huan Yu, Shao-Ming Fei

**Affiliations:** 1 School of Mathematical Sciences, Capital Normal University, Beijing 100037, P. R. China; 2 School of Mathematics and Statistics, Northeast Normal University, Jilin, Changchun 130024, P. R. China

## Abstract

Determining the quantum circuit complexity of a unitary operation is an important problem in quantum computation. By using the mathematical techniques of Riemannian geometry, we investigate the efficient quantum circuits in quantum computation with *n* qutrits. We show that the optimal quantum circuits are essentially equivalent to the shortest path between two points in a certain curved geometry of *SU*(3*^n^*). As an example, three-qutrit systems are investigated in detail.

Due to the quantum parallelism, quantum computers can solve efficiently problems that are considered intractable on classical computers^1^, e.g., algorithm for finding the prime factors of an integer^2^,^3^ and quantum searching algorithm^4^. A quantum computation can be described as a sequence of quantum gates, which determines a unitary evolution *U* performed by the computer. An algorithm is said to be efficient if the number of gates required grows only polynomially with the size of the problem. A central problem of quantum computation is to find efficient quantum circuits to synthesize desired unitary operation *U* used in such quantum algorithms.

A geometric approach to investigate such quantum circuit complexity for qubit systems has been developed in^5^,^6^,^7^. It is shown that the quantum circuit complexity of a unitary operation is closely related to the problem of finding minimal length paths in a particular curved geometry. The main idea is to introduce a Riemannian metric on the space of *n*-qubit unitary operations, chosen in such a way that the metric distance *d*(*I*, *U*) between the identity operation and a desired unitary *U* is equivalent to the number of quantum gates required to synthesize *U* under certain constraints. Hence the distance *d*(*I*, *U*) is a good measure of the difficulty of synthesizing *U*.

In fact, *d*-dimensional quantum states (qudits) could be more efficient than qubits in quantum information processing such as key distribution in the presence of several eavesdroppers. They offer advantages such as increased security in a range of quantum information protocols^8^,^9^,^10^,^11^,^12^, greater channel capacity for quantum communication^13^, novel fundamental tests of quantum mechanics^14^, and more efficient quantum gates^15^. In particular, hybrid qubit-qutrit system has been extensively studied and already experimentally realized^16^,^17^. The higher dimensional version of qubits provides deeper insights in the nature of quantum correlations and can be accessed by encoding qudits in the frequency modes of photon pairs produced by continuous parametric down-conversion.

In particular, the three-dimensional quantum states, qutrits are of special significance. For instance, in the state-independent experimental tests of quantum contextuality, three ground states of the trapped ^171^*Yb*^+^ ion are mapped to a qutrit system and quantum operations are carried out by applying microwaves resonant to the qutrit transition frequencies^18^. The solid-state system, nitrogen-vacancy center in diamond, can be also served as a qutrit system, in which the electronic spin can be individually addressed, optically polarized, manipulated and measured with optical and microwave excitation. Due to its long coherence time, it is one of the most promising solid state systems as quantum information processors.

In this paper we study the quantum information processing on qutrit systems. We generalize the results for qubit-systems^7^ to qutrit ones. The efficient quantum circuits in quantum computation with *n* qutrits are investigated in terms of the geometry of *SU*(3*^n^*). Three-qutrit systems are investigated in detail. Compared with the results for qubit systems^7^, our results are more fined, in the sense that by using enough one- and two-qutrit gates it is possible to synthesize a unitary operation with sufficient accuracy. While from^7^, it is not guaranteed that the error of the approximation would be arbitrary small.

## Results

A quantum gate on *n*-qutrit states is a unitary operator *U* ∈ *SU*(3*^n^*) determined by time-dependent Hamiltonian *H*(*t*) according to the Schr*ö*dinger equation, 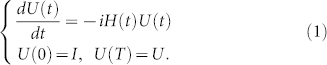
For qutrit case the Hamiltonian *H* can be expanded in terms of the Gell-Mann matrices. As the algebra related to the *n*-qutrit space has rather different properties from the qubits case in which the evolved Pauli matrices have very nice algebraic relations, we first present some needed results about the algebra *su*(3*^n^*).

Let *λ_i_*, *i* = 1, …, 8, denote the Gell-Mann matrices, 
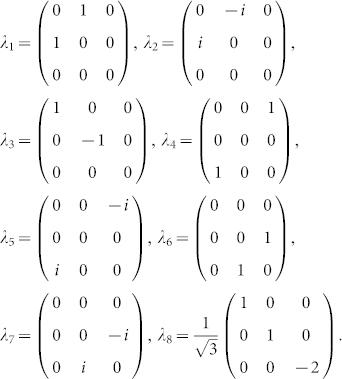


Let 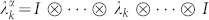
be an operator acting on the *α*th qutrit with *λ_k_* and the rest qutrits with identity *I*. The basis of *su*(3*^n^*) is constituted by {Λ*_s_*}, *s* = 1, …, *n*, where 
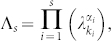
1 ≤ *α*_1_ < *α*_2_ < … < *α_s_* ≤ *n*, 1 ≤ *k_i_* ≤ 8. Λ*_s_* stands for all operators acting on *s* qutrits at sites *α*_1_, *α*_2_, …, *α_s_* with Gell-Mann matrices 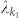, 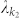, …, 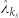 respectively, and the rest with identity. We call an element in {Λ*_s_*} an *s*-body one. By using the commutation relations among the Gell-Mann matrices, it is not difficult to prove the following conclusion:

**Lemma 1** All *s*-body items (*s* ≥ 3) in the basis of *su*(3*^n^*) can be generated by the Lie bracket products of 1-body and 2-body items.

In the following the operator norm of an operator *A* will be defined by 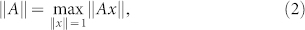
which is equivalent to the operator norm given by <*A*, *B*> = *tr A*^†^*B*. The norm of above Gell-Mann matrices satisfies ||*λ_i_*|| = 1, *i* = 1, …, 7, and 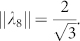. If we replace *λ*_8_ with 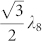, the Gell-Mann matrices are then normal with respect to the definition of the operator norm, and the basis of *su*(3*^n^*), still denoted by {Λ*_s_*}, is normalized.

A general unitary operator *U* ∈ *SU*(3*^n^*) on *n*-qutrit states can be expressed as *U* = *U*_1_*U*_2_…*U_k_* for some integer *k*. According to Lemma 1, every *U_i_* acts non-trivially only on one or two vector components of a quantum state vector, corresponding to a Hamiltonian *H_i_* containing only one and two-body items in {Λ*_s_*}, *s* = 1, 2.

The time-dependent Hamiltonian *H*(*t*) can be expressed as 
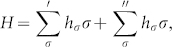
where: (1) in the first sum 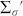, *σ* ranges over all possible one and two-body interactions; (2) in the second sum 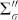, *σ* ranges over all other more-body interactions; (3) the *h_σ_* are real coefficients. We define the measure of the cost of applying a particular Hamiltonian in synthesizing a desired unitary operation *U*, similar to the qubit case, 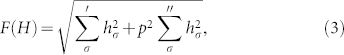
where *p* is the penalty paid for applying three- and more-body items.

Eq. (3) gives rise to a natural notion of distance in the space *SU*(3*^n^*) of *n*-qutrit unitary operators with unit determinant. A curve [*U*] between the identity operation *I* and the desired operation *U* is a smooth function, 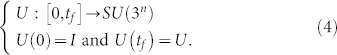
The length of this curve is given by 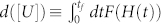. As d([U]) is invariant with respect to different parameterizations of [*U*], one can always set *F*(*H*(*t*)) = 1 by rescaling *H*(*t*), and hence *U* is generated at the time *t_f_* = *d*([*U*]). The distance *d*(*I*, *U*) between *I* and *U* is defined by 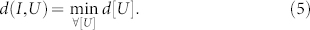
The function *F*(*H*) can be thought of as the norm associated to a right invariant Riemannian metric whose metric tensor *g* has components: 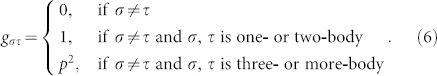
These components are written with respect to a basis for local tangent space corresponding to the coefficients *h_σ_*. The distance *d*(*I*, *U*) is equal to the minimal length solution to the geodesic equation, 〈*dH*/*dt*, *J*〉 = *i*〈*H*, [*H*, *J*]〉. Here 〈·, ·〉 is the inner product on the tangent space *su*(3*^n^*) defined by the above metric components, and *J* is an arbitrary operator in *su*(3*^n^*).

From Lemma **1** in the basis {Λ*_s_*} of *su*(3*^n^*), all the *q*-body items (*q* ≥ 3) can be generated by Lie bracket products of 1-body and 2-body items. To find the minimal length solution to the geodesic equation, it is reasonable to choose such metric (6), because the influence of there- and more-body items will be ignorable for sufficiently large *p*. It is the one- and two-body items that mainly contribute to the minimal geodesic.

We first project the Hamiltonian *H*(*t*) onto *H_P_*(*t*) which contains only one- and two-qutrit items. By choosing the penalty *p* large enough we can ensure that the error in this approximation is small. We then divide the evolution according to *H_P_*(*t*) into small time intervals and approximate with a constant mean Hamiltonian over each interval. We approximate evolution according to the constant mean Hamiltonian over each interval by a sequence of one- and two-qutrit quantum gates. We show that the total errors introduced by these approximations can be made arbitrarily smaller than any desired constant.

Let *M* be a connected manifold and 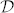 a connection on a principal *G*-bundle. The Chow's theorem^19^ says that the tangent space *M_q_* at any point *q* ∈ *M* can be divided into two parts, the horizontal space *H_q_M* and the vertical space *V_q_M*, where 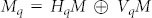 and 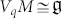 (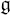 denotes the Lie algebra of G). Let 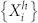 be a local frame of *H_q_M*. Then any two points on *M* can be joined by a horizontal curve if the iterated Lie brackets 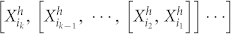 evaluated at *q* span the tangent space *M_q_*.

**Lemma 2** Let *p* be the penalty paid for applying three- and more-body items. If one chooses *p* to be sufficiently large, the distance *d*(*I*, *U*) always has a supremum which is independent of *p*.

### Proof

As *SU*(3*^n^*) is a connected and complete manifold, the tangent space at the identity element *I* can be looked upon as the Lie algebra *su*(3*^n^*). For a given right invariant Riemannian metric (6), there exists a unique geodesic joining *I* and some point *U* ∈ *SU*(3*^n^*). With the increase of *p*, the distance *d*(*I*, *U*, *p*) of the geodesic joining *I* and *U* ∈ *SU*(3*^n^*) increases monotonically.

On the other hand, according to Lemma **1**, 1-body and 2-body items in the basis {Λ*_s_*} can span the whole space *su*(3*^n^*) in terms of the Lie bracket iterations. Under the metric Eq.(6), from the Chow's theorem we have that the horizontal curve joining *I* and *U* ∈ *SU*(3*^n^*) is unique, since the subspace spanned by 1-body and 2-body items is invariable. Or there exists such a geodesic that its initial tangent vector lies in the subspace spanned by 1-body and 2-body items. Hence the distance *d*(*I*, *U*, *p*) has a sup *d*_0_ which is independent of *p*.

**Lemma 3** Let *H_P_*(*t*) be the projected Hamiltonian containing only one- and two-body items, obtained from a Hamiltonian *H*(*t*) generating a unitary operator *U*, and *U_P_* the corresponding unitary operator generated by *H_P_*(*t*). Then 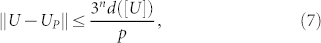
where || · || is the operator norm defined by (2), and *p* is the penalty parameter in (6).

### Proof

Let *U* and *V* be unitary operators generated by the time-dependent Hamiltonians *H*(*t*) and *J*(*t*) respectively, 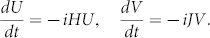


By integrating above two equations in the interval [0, *T*], we have 
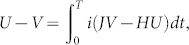
where *U*(*T*) = *U*, *V*(*T*) = *V* and *U*(0) = *V*(0) = *I* have been taken into account.

Since 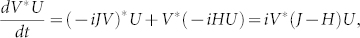
we have 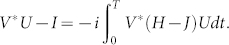


Using the triangle inequality and the unitarity of the operator norm || · ||, we obtain: 
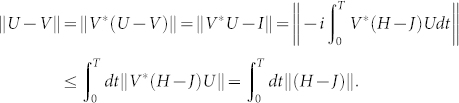


The Euclidean norm of the Hamiltonian 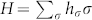 is given by 
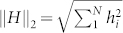
. From the Cauchy-Schwarz inequality, we have 1
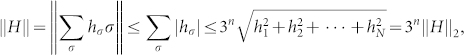
Moreover, if *H* contains only three- and more-body items, we have 
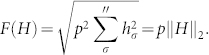


Therefore 
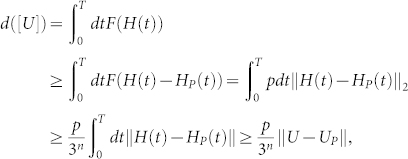
which gives rise to (7).

### Remark

From Lemma 3, by choosing *p* sufficiently large, say *p* = 9*^n^*, we can ensure that ||*U* − *U_P_*|| ≤ *d*([*U*])/3*^n^*. Moreover, since the distance *d*(*I*, *U*) is defined by *d*(*I*, *U*) = min_µ[*U*]_*d*[*U*], Lemma **3** also implies that ||*U* − *U_P_*|| ≤ *d*(*I*, *U*)/3*^n^*.

**Lemma 4** If *U* is an *n*-qutrit unitary operator generated by *H*(*t*) satisfying ||*H*(*t*)|| ≤ *c* in a time interval [0, Δ], then 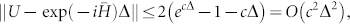
where 
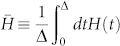
 is the mean Hamiltonian.

### Proof

Recall the Dyson series^20^: 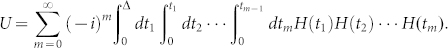


We choose *t_i_* ≤ Δ/(*i* + 1) and set the first term in the above series to be I. Hence the second term is 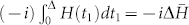. We have 
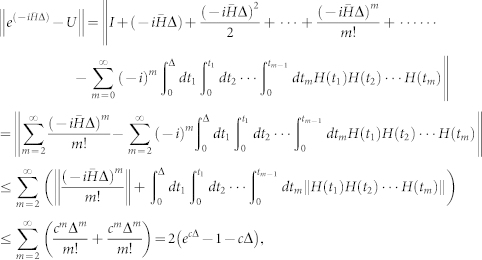
where we have used the standard norm inequality ||*XY*|| ≤ ||*X*|| ||*Y*||, the condition ||*H*(*t*)|| ≤ *c*, 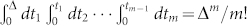 and 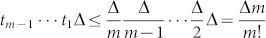.

**Proposition 1** If *A* and *B* are two unitary operators, then 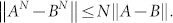


### Proof

We begin with *N* = 2. It is easy to verify that 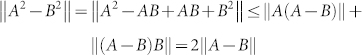


Now suppose that this inequality holds for *N* − 1, *N* ≥ 3, i.e., ||*A^N^*^−1^ − *B^N^*^−1^|| ≤ (*N* − 1)||*A* − *B*||. Then for *N* we have 
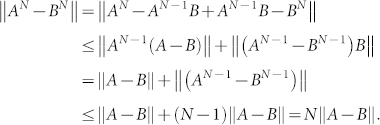


**Lemma 5** Suppose *H* is an *n*-qutrit one- and two-body Hamiltonian whose coefficients satisfy |*h_σ_*| ≤ 1. Then there is a unitary operator *U_A_* which satisfies 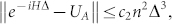
and can be synthesized by using at most *c*_1_*n*^2^/Δ one- and two-tribit gates, where *c*_1_ and *c*_2_ are constants.

### Proof

We need a modified version of the Trotter formula^1^: let A and B be Hermitian operators, then *e^i^*^(*A*+*B*)Δ*t*^ = *e^iA^*^Δ*t*^*e^iB^*^Δ*t*^ + *O*(Δ*t*^2^). We divide the interval [0, Δ] into *N* = 1/Δ intervals of size Δ^2^. In every interval, we define a unitary operator 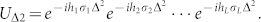


There are *L* = 32*n*^2^ − 24*n* = *O*(*n*^2^) one- and two-body items in *H*. From the modified Trotter formula, there exists a constant *c*_2_ such that 
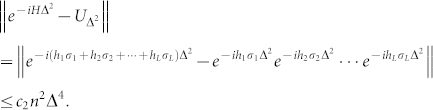


By using Proposition **1**, we have 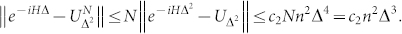


It means that one can approximate *e*^−*iH*Δ^ by using at most *Nc*_1_*n*^2^ = *c*_1_*n*^2^/Δ quantum gates for some constant *c*_1_.

From the above we have our main result:

**Theorem 1** Using *O*(*n^K^d*(*I*, *U*)^3^) (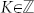) one- and two-qutrit gates it is possible to synthesize a unitary *U_A_* satisfying ||*U* − *U_A_*|| ≤ *c*, where *c* is any constant.

Theorem 1 shows that the optimal way of generating a unitary operator in *SU*(3*^n^*) is to go along the minimal geodesic curve connecting I and U. As an detailed example, we study the three-qutrit systems. In this case the right invariant Riemannian metric (6) turns out to be a more general one^21^, 
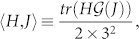
where 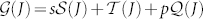, *p* is the penalty parameter and *s* is the parameter meaning that one-body Hamiltonians may be applied for free when it is very small, 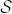 maps the three-qutrit Hamiltonian to the subspace containing only one-body items, 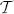 to the subspace containing only two-body items, and 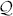 to the subspace containing only three-body items. According to the properties of the Gell-Mann matrices, they satisfy 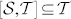, 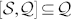, 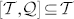.

Set 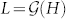, 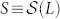, 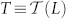 and 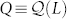. From the geodesic equation 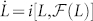, where 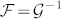, we have 
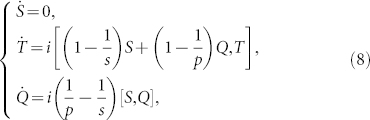
which gives rise to the solution 
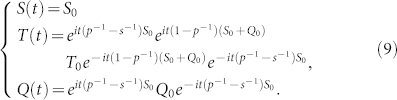
where *S*(0) = *S*_0_, *T*(0) = *T*_0_ and *Q*(0) = *Q*_0_.

The corresponding Hamiltonian 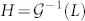 has the form: 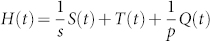. According to the assumption 〈*H*(*t*), *H*(*t*)〉 = 1 for all time *t*, we have 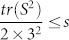, 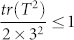, and 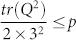. The term 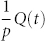 in *H*(*t*) is of order *p*^−1/2^, and hence can be neglected in the large *p* limit, with an error of order *tp*^−1/2^. Also the term containing *p*^−1^ in the exponentials of *T* can be neglected with an error at most of order *t*^2^(*s*^1/2^*p*^−1^ + *p*^−1/2^). Therefore one can define an approximate Hamiltonian 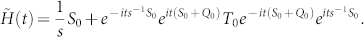


The corresponding solution 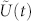 of the Schr*ö*dinger equation satisfies 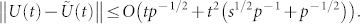


Denote 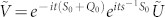. Then 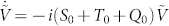 and 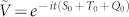. Thus we have 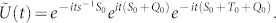


Generally one can expect that *S*_0_ + *Q*_0_ is much lager than *T*_0_, and *S*_0_ + *Q*_0_ is nondegenerate. 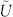 can be simplified at the first-order perturbation, 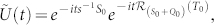
where 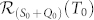 denotes the diagonal matrix by removing all the off-diagonal entries from *T*_0_ in the eigenbasis of *S*_0_ + *Q*_0_. Therefore we see that it is possible to synthesize a unitary 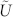 satisfying 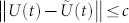, where *c* is any constant, say *c* = 1/10.

## Discussion

We have investigated the efficient quantum circuits in quantum computation with *n* qutrits in terms of Riemannian geometry. We have shown that the optimal quantum circuits are essentially equivalent to the shortest path between two points in a certain curved geometry of *SU*(3*^n^*), similar to the qubit case where the geodesic in *SU*(2*^n^*) is involved^7^. As an example, three-qutrit systems have been investigated in detail. Some algebraic derivations involved for qutrit systems are rather different from the ones in qubit systems. In particular, we used (2) as the norm of operators. The operator norm of *M* used in^7^ is defined by ||*M*||_1_ = max_〈*ψ*|*ψ*〉 = 1_{|〈*ψ*|*M*|*ψ*〉|}, which is not unitary invariant in the sense that ||*M*||_1_ = ||*U M*||_1_ = ||*M U*||_1_ is not always true for any unitary operator *U*. For instance, consider 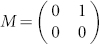 and 
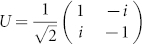
. One has ||*M*||_1_ = 1/2. However, 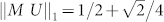. Generally, from Cauchy-Schwarz inequality one has ||*M*||_1_ ≤ ||*M*||. If *M*^†^*M* = *I* or *M*^†^ = *M*, then ||*M*||_1_ = ||*M*||.

Moreover, the final results we obtained are finer than the ones in^7^. Our result shows that if *k* in formula 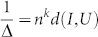 is taken to be sufficiently large, ||*U* − *U_A_*|| can be sufficiently small. However, the approximation error estimation in^7^ reads 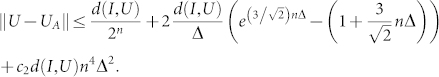


First, since *d*(*I*, *U*) is dependent of the penalty parameter *p*, there should exist a *p*-independent bound to guarantee that 2*^n^d*(*I*, *U*)/*p* is small for sufficiently large *p*. Second, if one chooses Δ as scale 1/*n*^2^*d*(*I*, *U*), the sum of the last two terms of the right hand side is 9/2 + *c*_2_/*d*(*I*, *U*) + O. Therefore the scale should be smaller, for example, 1/*n^k^d*(*I*, *U*) and *k* > 3. As Δ takes the scale of 1/*n*^2^*d*(*I*, *U*) in^7^, it can not guarantee that the error in the approximation could be arbitrary small.

Due to the special properties of the Pauli matrices involved in qubit systems, many derivations for qubit systems are different from the ones for qutrit systems. Nevertheless, the derivations for qutrit systems in this paper can be generalized to general high dimensional qudit systems.

## Methods

In deriving Theorem 1, we use Lemmas 2-5. Let *H*(*t*) be the time-dependent normalized Hamiltonian generating the minimal geodesic of length *d*(*I*, *U*). Let *H_P_*(*t*) be the projected Hamiltonian which contains only the one- and two-body items in *H*(*t*) and generates *U_P_*. According to Lemma 3, they satisfy 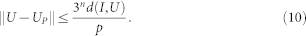
Divide the time interval [0, *d*(*I*, *U*)] into *N* parts with each of length Δ = *d*(*I*, *U*)/*N*. Let 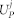 be the unitary operator generated by *H_P_*(*t*) in the *j*th time interval, and 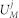 be the unitary operator generated by the mean Hamiltonian 
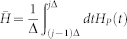
. Then using Lemma 4 and inequality 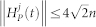 we have 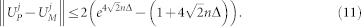
As *F*(*H*) is scaled to be one, 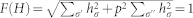, one has 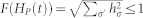. Hence 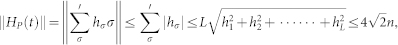
where *L* = 32*n*^2^ – 24*n* is the number of one- and two-body items in *H*(*t*), i.e. the number of the terms in *H_P_*(*t*)).

Applying Lemma 5 to 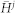 on every time interval, we have that there exists a unitary 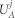 which can be synthesized by using at most *c*_1_*n*^2^/Δ one- and two-qutrit gates, and satisfies 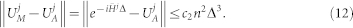
*U_P_* and *U_A_* can be generated in terms of 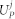 and 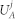, respectively. We show how to generate *U_P_* by use of 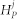 below. First, 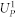 can be generated by 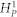: 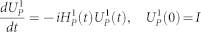
with 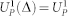. The unitary operator 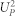 generated by 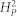 satisfies 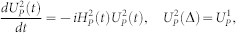
which can be transformed into 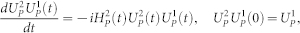
with 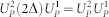, where 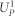 is constant in [Δ, 2Δ]. At last we have 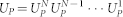 generated by the Hamiltonians 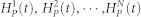. *U_A_* can be generated similarly.

Therefore 
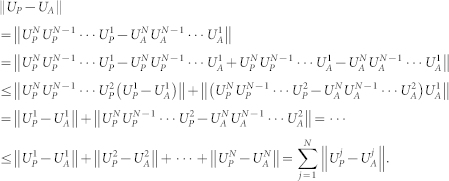


From (10), (11) and (12) we obtain: 
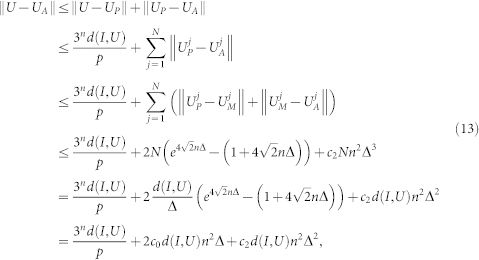
where 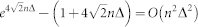 and *c*_0_ is a constant.

As mentioned in Lemma 2, the distance *d*(*I*, *U*) has a sup *d*_0_ for sufficiently large *p*. For example, we choose a suitable penalty *p* so that *d*(*I*, *U*, *p*) satisfies 
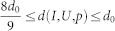
. If we choose Δ to be sufficiently small, e.g. 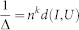 with *k* sufficiently large, ||*U* – *U_A_*|| will be sufficiently small, 
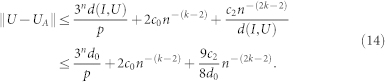
As we need *c*_1_*n*^2^/Δ one- and two-body gates to synthesize every 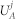, we ultimately need 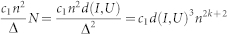 one- and two-body gates.

## Author Contributions

B.L. and Z.H. and S.M. wrote the main manuscript text. All authors reviewed the manuscript.
